# Actomyosin contractility provokes contact inhibition in E-cadherin-ligated keratinocytes

**DOI:** 10.1038/srep46326

**Published:** 2017-04-13

**Authors:** Hiroaki Hirata, Mikhail Samsonov, Masahiro Sokabe

**Affiliations:** 1 R-Pharm Japan, Tokyo 105-0001, Japan; 2 Mechanobiology Laboratory, Nagoya University Graduate School of Medicine, Nagoya 466-8550, Japan; 3 R-Pharm, Moscow 123317, Russia

## Abstract

Confluence-dependent inhibition of epithelial cell proliferation, termed contact inhibition, is crucial for epithelial homeostasis and organ size control. Here we report that among epithelial cells, keratinocytes, which compose the stratified epithelium in the skin, possess a unique, actomyosin-dependent mechanism for contact inhibition. We have observed that under actomyosin-inhibited conditions, cell-cell contact itself through E-cadherin promotes proliferation of keratinocytes. Actomyosin activity in confluent keratinocytes, however, inhibits nuclear localization of β-catenin and YAP, and causes attenuation of β-catenin- and YAP-driven cell proliferation. Confluent keratinocytes develop E-cadherin-mediated punctate adhesion complexes, to which radial actin cables are connected. Eliminating the actin-to-E-cadherin linkage by depleting α-catenin increases proliferation of confluent keratinocytes. By contrast, enforced activation of RhoA-regulated actomyosin or external application of pulling force to ligated E-cadherin attenuates their proliferation, suggesting that tensile stress at E-cadherin-mediated adhesion complexes inhibits proliferation of confluent keratinocytes. Our results highlight actomyosin contractility as a crucial factor that provokes confluence-dependent inhibition of keratinocyte proliferation.

Tight regulation of the cell number in epithelial tissues is essential for epithelial integrity and organ size control^1^,^2^. The epithelial cell density is maintained through various mechanisms including division and differentiation of stem and progenitor cells^3^,^4^, cell competition^5^,^6^, and cell extrusion^7^. Confluence-dependent arrest of cell proliferation, termed contact inhibition, is another major strategy for homeostatic control of the cell density^8^. While most solid tumors originate from epithelia, loss of contact inhibition is a typical hallmark of cancer cells and contributes to their overgrowth and tumorigenesis, which highlights a critical role of contact inhibition in epithelial homeostasis^8^,^9^.

The prototypical epithelial cadherin E-cadherin mediates Ca^2+^-dependent adhesion between epithelial cells. The role of E-cadherin homophilic binding in inducing contact inhibition of epithelial cell proliferation has been appreciated^10^,^11^,^12^,^13^. On the other hand, recent studies have unveiled that various mechanical cues, including actomyosin activity^14^,^15^, individual cell areas^16^,^17^, and stiffness^18^, strain^17^,^19^ and topography^20^ of extracellular substrates, also affect proliferation of confluent epithelial cells. Since all these mechanical cues tune cytoskeletal tension, potential involvement of the tension in the regulation of epithelial cell proliferation has been discussed^16^,^17^,^21^. However, molecular and architectural bases underlying the tension-dependent proliferation regulation are largely unclear. Furthermore, it is elusive how E-cadherin ligation and cytoskeletal tension cooperate to achieve contact inhibition of epithelial cell proliferation.

Keratinocytes form epithelial layers in the epidermis, providing a stable environmental barrier in the skin^22^. They exhibit typical contact inhibition in their proliferation^23^,^24^. Although keratinocytes proliferate exclusively at the basal layer in normal epidermis, loss of contact inhibition causes their proliferation even in supra-basal layers, which is associated with formation of disorganized keratinocyte masses^23^. Therefore, contact inhibition of keratinocyte proliferation is crucial for epidermal homeostasis. Keratinocytes in epidermis contact each other not only at their lateral surfaces but also at their apical and basal surfaces, which stands in contrast to the case of the epithelial cells forming simple, mono-layered epithelia. Thus, biochemical and mechanical environments surrounding contact-inhibited epithelial cells are largely different between epidermis and simple, mono-layered epithelia. Despite this, it has not been asked whether or not keratinocytes adopt the same mechanism for contact inhibition as the one that is employed by epithelial cells of mono-layered epithelia.

Here, we report that actomyosin activity is required to provoke contact inhibition in human HaCaT keratinocytes, but not in human mammary gland MCF-10A epithelial cells. Results suggest that while E-cadherin ligation itself promotes proliferation of keratinocytes, actomyosin-based tension at E-cadherin-mediated cell-cell adhesion complexes inhibits keratinocyte proliferation.

## Results and Discussion

**Figure 1:**
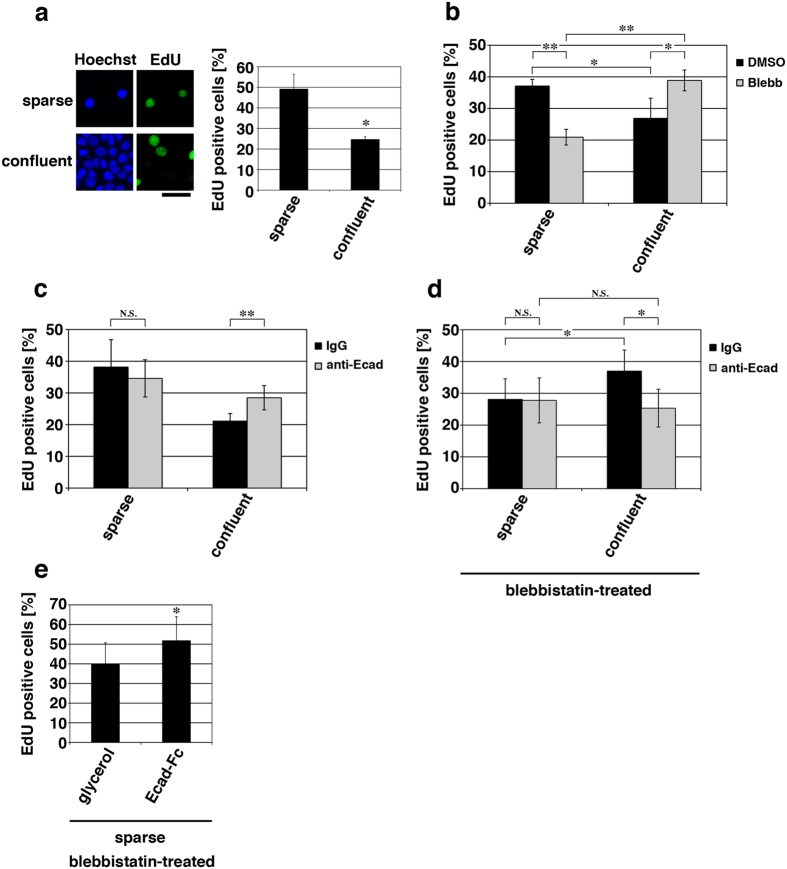
E-cadherin ligation promotes, but actomyosin activity inhibits proliferation of confluent keratinocytes. (**a**) HaCaT cells were cultured for 40 h after seeding the cells at the sparse (0.3 × 10^4^ cells/cm^2^) or the confluent (10 × 10^4^ cells/cm^2^) cell density, and their proliferation was evaluated by EdU incorporation during a 2-h incubation. Total nuclei were labeled with Hoechst. Scale bar, 50 μm. The bar graph shows the percentages of EdU-positive cells. *P < 0.005. *n* = 8 (>50 cells each) for each bar. (**b**–**d**) Cells cultured for 40 h under the sparse and confluent conditions were treated with 100 μM blebbistatin (Blebb) (**b**), 5 μg/ml anti-E-cadherin inhibitory antibody (anti-Ecad) (**c**) or 100 μM blebbistatin (Blebb) together with 5 μg/ml anti-E-cadherin inhibitory antibody (anti-Ecad) (**d**) for 6 h. As controls, cells were treated with either DMSO (**b**) or control IgG (**c** and **d**). The cells were then incubated with EdU for 2 h in the presence of these drugs. The percentages of EdU positive cells are shown. *P < 0.05; **P < 0.005; N.S., no significant difference. *n* = 8 (>50 cells each) for each bar. (**e**) Cells cultured for 40 h under the sparse condition were treated with 100 μM blebbistatin (Blebb) together with 40 μg/ml E-cadherin-Fc (Ecad-Fc) or glycerol (control) for 6 h, and then incubated with EdU for 2 h in the presence of blebbistatin and either E-cadherin-Fc or glycerol. The percentages of EdU positive cells are shown. *P < 0.05. *n* = 8 (>50 cells each) for each bar.

When cell proliferation was monitored by EdU incorporation, human HaCaT keratinocytes cultured for 40 h after seeding the cells showed confluence-dependent inhibition of proliferation^24^,^25^ (Fig. 1a). EdU positive cells were also positive in cyclin E expression (Supplementary Fig. S1a), indicating S-phase entry and progression in these cells. To investigate the role of actomyosin activity in keratinocyte proliferation, myosin II ATPase activity was inhibited with blebbistatin. Blebbistatin treatment reduced cell proliferation in sparse cultures (Fig. 1b), which was consistent with the previous reports using different cell types^26^,^27^,^28^. By contrast, the ratio of EdU positive cells against total cells in confluent cultures was increased in response to the treatment with blebbistatin (Fig. 1b) or the Rho kinase inhibitor, Y-27632 (Supplementary Fig. S1b). While blebbistatin treatment caused a failure in cell division^29^ (Supplementary Fig. S1c), the frequency of cytokinetic events (including incomplete cell division) was increased upon the blebbistatin treatment (Supplementary Fig. S1d and Supplementary Videos 1 and 2). These results indicate that actomyosin activity has an inhibitory effect on cell cycle progression in confluent keratinocytes.

Contact inhibition of HaCaT cell proliferation was gradually established even when cells were seeded at the confluent cell density; ~50 h were required for achieving nearly complete inhibition of cell proliferation (Supplementary Fig. S2a). Blebbistatin treatment of HaCaT cells at the time point of 40 h after seeding the cells abrogated contact inhibition almost completely (Fig. 1b). By contrast, blebbistatin treatment at 64 h increased cell proliferation only slightly (Supplementary Fig. S2b). These results suggest that while actomyosin activity has an inhibitory effect on proliferation of confluent keratinocytes, additional mechanism(s) is likely to be involved in keeping cell proliferation arrested after contact inhibition is fully established. In this study, we focus on the role of actomyosin activity in the regulation of confluent keratinocyte proliferation during progression of contact inhibition (i.e., at 40 h after seeding cells).

In contrast to the case of HaCaT keratinocytes, proliferation of the human mammary gland epithelial cells (MCF-10A cells) was not affected by the blebbistatin treatment (Supplementary Fig. S2d), even though HaCaT and MCF-10A cells exhibited similar time courses of contact inhibition progression (Supplementary Fig. S2a,c).

Surprisingly, under myosin II inhibition, the ratio of EdU positive cells against total cells was higher in confluent keratinocyte cultures compared with that in sparse cultures (Fig. 1b). This implies that cell-cell contact formation in the absence of actomyosin activity may promote proliferation of keratinocytes. To test this possibility, we examined the effect of E-cadherin ligation on the proliferation of HaCaT cells. As expected from the role of E-cadherin ligation in contact inhibition of keratinocyte proliferation^12^,^30^, inhibition of E-cadherin homophilic ligation alone using the anti-E-cadherin inhibitory antibody increased proliferation of confluent HaCaT cells, whilst it did not affect proliferation of sparse cells (Fig. 1c). By contrast, the confluence-dependent increase in proliferation of myosin II-inhibited cells (Fig. 1b) was abrogated upon inhibition of E-cadherin ligation (Fig. 1d). This indicates that E-cadherin ligation is required for increasing proliferation of myosin II-inhibited cells under the confluent condition. We further tested whether E-cadherin ligation was sufficient for promoting keratinocyte proliferation under myosin II inhibition. When the recombinant E-cadherin-Fc chimeric protein, which acts as a functional E-cadherin ligand^31^, was applied to sparse cultures of myosin II-inhibited HaCaT cells, we observed an increase in cell proliferation (Fig. 1e). Taken together, our results indicate that E-cadherin ligation in the absence of actomyosin activity promotes proliferation of keratinocytes, and actomyosin activity is required for causing confluence-dependent inhibition of their proliferation. It is noteworthy that E-cadherin ligation was involved in confluence-dependent inhibition of proliferation of cells with normal actomyosin activity (Fig. 1c), which suggests that actomyosin activity alone without E-cadherin ligation is not sufficient for inhibiting proliferation of confluent keratinocytes. Thus, E-cadherin ligation is likely to be a prerequisite for actomyosin-dependent inhibition of keratinocyte proliferation under the confluent condition.

**Figure 2:**
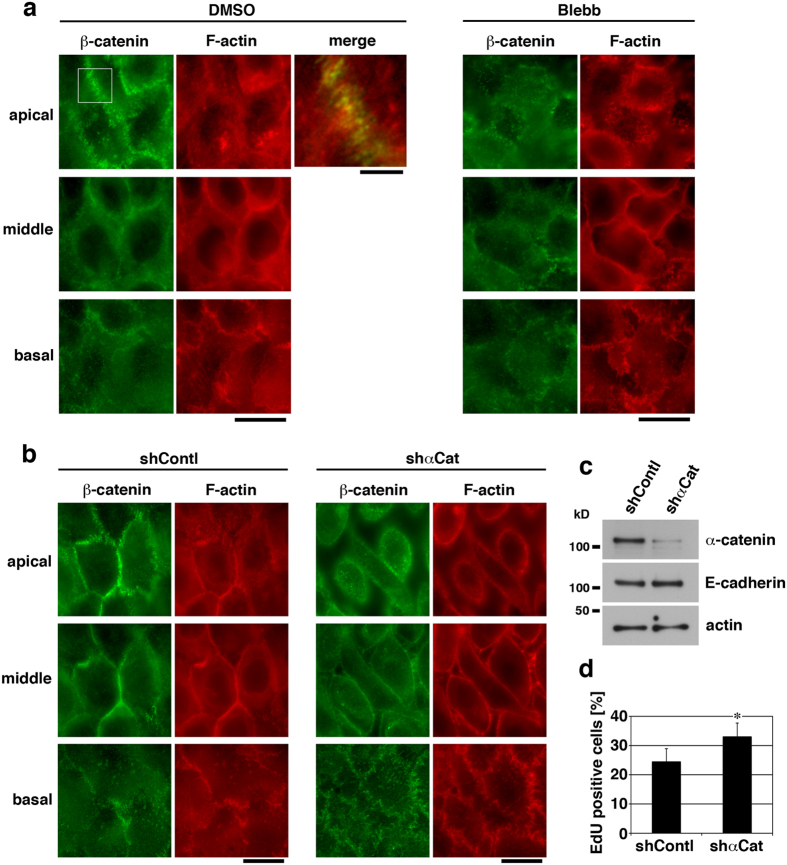
Role of apical adherens junctions in proliferation of confluent keratinocytes. (**a** and **b**) Wild-type HaCaT cells (**a**) or HaCaT cells expressing shRNA against α-catenin (shαCat) or non-targeting shRNA (shContl) (**b**) were cultured for 40 h under the confluent condition. Wild-type cells were then treated with 100 μM blebbistatin (Blebb) or DMSO (for control) for 6 h. These cells were stained for β-catenin and F-actin. Apical, middle and basal focal planes of the cells are shown. A magnified and merged image of the boxed region (DMSO, apical in **a**) is also shown. Scale bars, 5 μm for the magnified and merged image, and 20 μm for others. (**c**) Confluent HaCaT cells expressing shRNA against α-catenin (shαCat) or non-targeting shRNA (shContl), which were cultured for 40 h after the cell seeding, were lysed and immunoblotted for α-catenin, E-cadherin and actin. (**d**) Confluent HaCaT cells expressing shRNA against α-catenin (shαCat) or non-targeting shRNA (shContl) were cultured for 40 h and then incubated with EdU for 2 h. The percentages of EdU positive cells are shown. *P < 0.01. *n* = 8 (>50 cells each) for each bar.

Homophilically ligated E-cadherin forms cell-cell adhesion complexes, namely adherens junctions (AJs), to which the actin cytoskeleton is connected. Actomyosin-generated tensile force is transmitted to AJs and regulates their development^32^,^33^,^34^. Thus, AJs would provide an ideal platform for integrating signals of E-cadherin ligation and actomyosin activity in confluent cells. Therefore, we next asked whether AJs, in particular tensile force acting on AJs, play a role in E-cadherin- and actomyosin-dependent inhibition of proliferation of confluent keratinocytes. Confluent HaCaT cells developed prominent, punctate AJs at apical portions of cell-cell boundaries, and actin cables oriented perpendicularly against the boundaries were connected to these AJs (Fig. 2a and Supplementary Fig. S3a). Both E-cadherin ligation (Supplementary Fig. S3b) and myosin II activity (Fig. 2a and Supplementary Fig. S3a) were required for maintaining the apical AJs and the connected actin cables. In contrast to HaCaT cells, MCF-10A cells, which showed contact inhibition independently of actomyosin activity (Supplementary Fig. S2d), did not develop actomyosin-dependent punctate AJs, but formed continuous AJs that were resistant against actomyosin inhibition (Supplementary Fig. S4).

To examine the role of tensile force transmission from the actin cytoskeleton to AJs in keratinocyte proliferation, expression of α-catenin, a major linker between the actin cytoskeleton and the E-cadherin-β-catenin complexes at AJs^33^,^34^, was depleted using shRNA (Fig. 2b,c). Although α-catenin-depleted confluent HaCaT cells contacted each other with β-catenin localization at basal and middle portions of cell-cell boundaries, these cells did not form β-catenin-rich apical AJs and showed gaps between cells in apical regions (Fig. 2b). Associated with this, cell proliferation in confluent cultures was elevated upon depletion of α-catenin expression (Fig. 2d), which is consistent with the previous reports^23^,^35^,^36^. This result suggests that cell-cell contacts themselves are not enough for inhibiting proliferation of confluent keratinocytes, and the actin-AJ connection is required.

**Figure 3:**
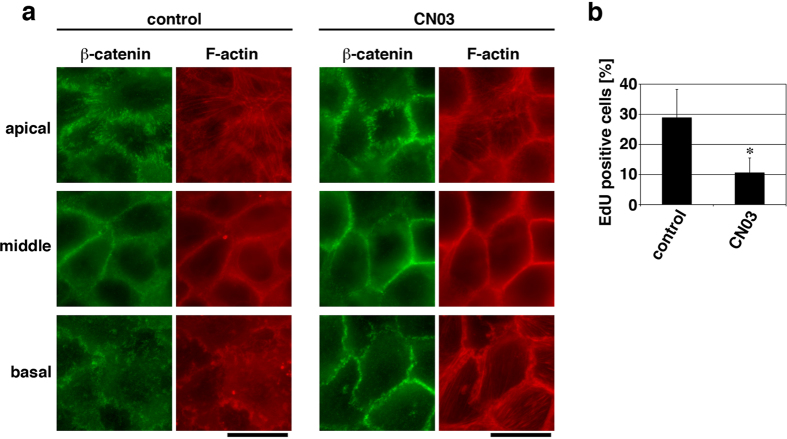
Enforced activation of RhoA in confluent keratinocytes develops adherens junctions and attenuates cell proliferation. (**a** and **b**) Confluent HaCaT cells cultured for 40 h were treated with or without 5 μg/ml CN03 for 6 h and then subjected to either immunostaining for β-catenin and F-actin (**a**) or the EdU incorporation assay (**b**). Apical, middle and basal focal planes of the cells are shown in (**a**). Scale bars, 20 μm. The percentages of EdU positive cells are shown in (**b**). *P < 0.005. *n* = 8 (>50 cells each) for each bar.

The inhibitory role of actomyosin activity in proliferation of confluent keratinocytes was further examined by testing whether an enforced elevation of actomyosin activity in confluent keratinocytes caused a reduction in cell proliferation. To this end, endogenous RhoA was activated using the membrane-permeable RhoA activator CN03, which is derived from the bacterial cytotoxic necrotizing factor and constitutively activates RhoA by deamidating Gln63 of RhoA^37^,^38^. Although treatment of confluent HaCaT cells with CN03 did not apparently affect organizations of β-catenin and F-actin in apical and middle regions of the cells, it caused substantial development of AJs (with β-catenin localization and F-actin accumulation along cell-cell boundaries) at the basal portions of cell-cell boundaries (Fig. 3a). Associated with this, cell proliferation under the confluent condition was decreased upon the CN03 treatment (Fig. 3b), which is consistent with the notion that actomyosin-based force at AJs has an inhibitory effect on proliferation of confluent keratinocytes. By contrast, CN03 treatment of confluent MCF-10A epithelial cells, which caused development of apical actin cables and basal stress fibers (Supplementary Fig. S5a), did not affect their proliferation (Supplementary Fig. S5b), suggesting again that proliferation of confluent MCF-10A cells is actomyosin-independent.

**Figure 4:**
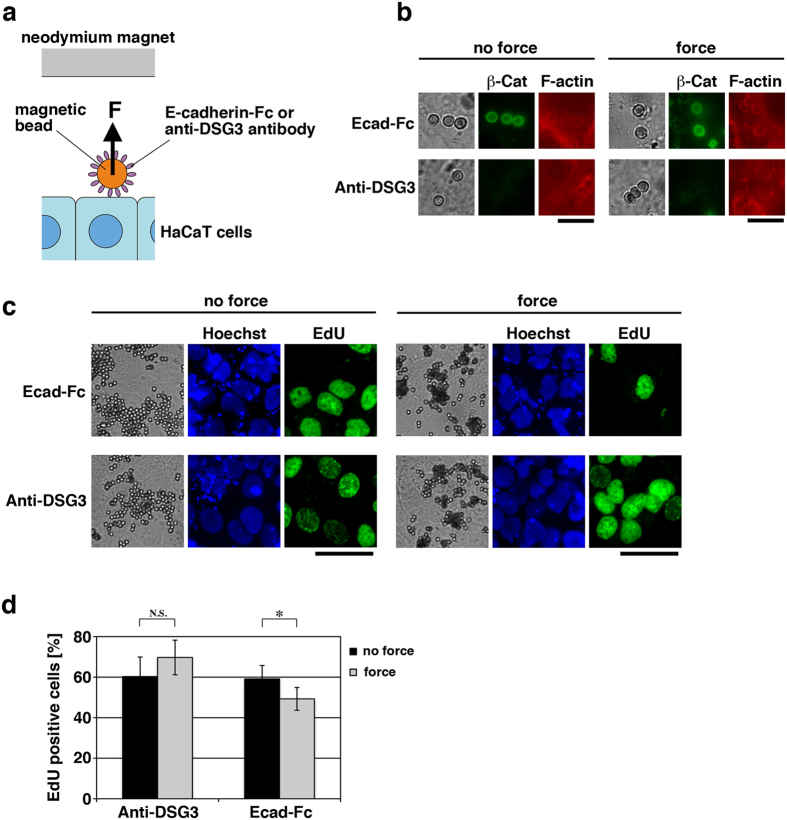
Application of tensile force to ligated E-cadherin attenuates proliferation of actomyosin-inhibited keratinocytes. (**a**) A schematic drawing of the method for tensile force application through ligated E-cadherin onto HaCaT cells. E-cadherin-Fc or the anti-desmoglein 3 (DSG3) antibody was covalently coupled to protein G-conjugated magnetic beads. Pulling force (F) was applied to the beads attached to the cells by placing a neodymium magnet above the cell culture dish. (**b**–**d**) Confluent HaCaT cells cultured for 40 h were first incubated for 1 h with E-cadherin-Fc (Ecad-Fc)- or anti-desmoglein 3 antibody (Anti-DSG3)-conjugated magnetic beads in the presence of 100 μM blebbistatin. After washing free beads out, the cells were further incubated in the presence of blebbistatin for 6 h with (force) or without (no force) neodymium magnets placed above the cell culture dishes. The cells were then subjected to either immunostaining for β-catenin and F-actin (**b**) or the EdU incorporation assay (**c** and **d**). Beads were shown in bright field images, and total nuclei were stained with Hoechst. Scale bars, 10 μm in (**b**) and 40 μm in (**c**). The percentages of EdU positive cells are shown in (**d**). *P < 0.05; N.S., no significant difference. *n* = 8 (>50 cells each) for each bar.

To dissect the role of tensile force at AJs more directly, we externally applied tensile force through homophilic E-cadherin bonds to AJs in myosin II-inhibited cells using magnetic beads conjugated with E-cadherin-Fc (Fig. 4a). When confluent HaCaT cells were incubated with the beads in the presence of blebbistatin, β-catenin and F-actin were accumulated around E-cadherin-Fc-conjugated beads contacted to the cell surfaces, but not around the beads conjugated with the antibody against a non-AJ membrane protein (desmoglein 3; DSG3) (Fig. 4b). Application of magnetic force through the E-cadherin-Fc-conjugated beads caused a decrease in cell proliferation, whilst force application through the anti-DSG3 antibody-conjugated beads did not (Fig. 4c,d). This suggests that tensile force acting on E-cadherin-mediated AJs attenuates proliferation of confluent keratinocytes.

**Figure 5:**
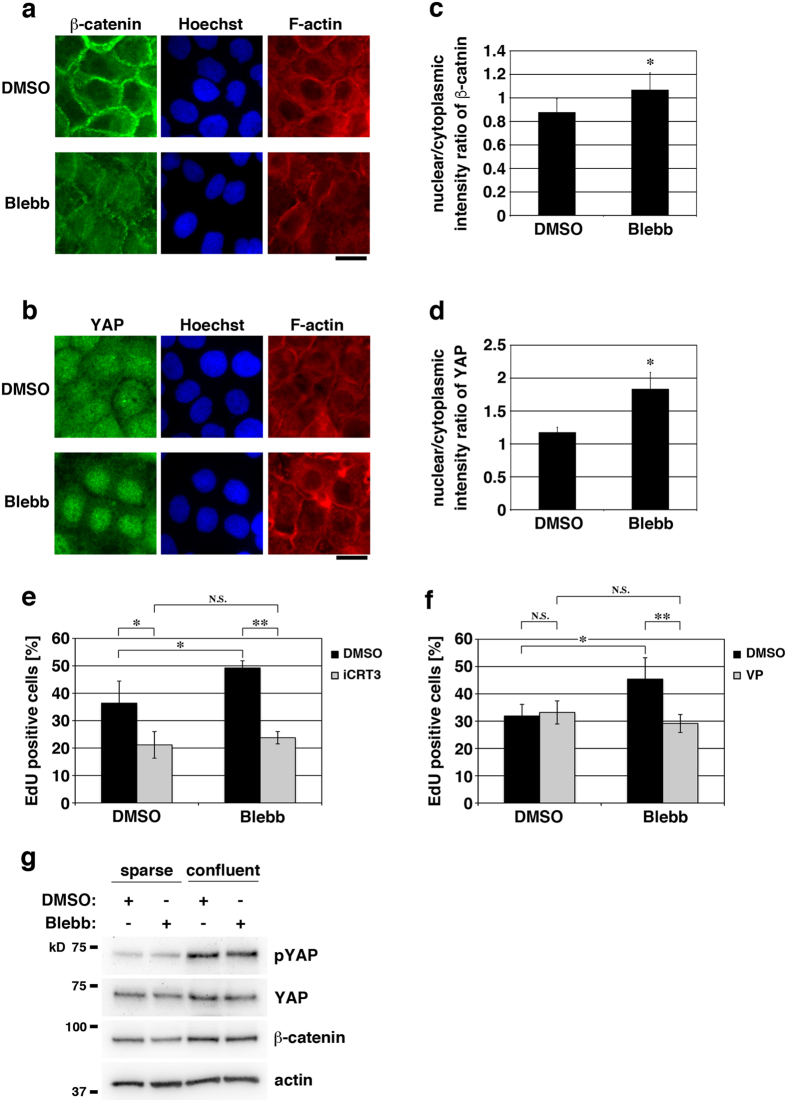
Actomyosin activity inhibits β-catenin- and YAP-driven proliferation of confluent keratinocytes. (**a** and **b**) Confluent HaCaT cells cultured for 40 h were treated with 100 μM blebbistatin (Blebb) or DMSO (for control) for 6 h, and then stained for nuclei (Hoechst), F-actin and either β-catenin (**a**) or YAP (**b**). Scale bars, 20 μm. (**c** and **d**) The nuclear/cytoplasmic ratio of fluorescence intensities of β-catenin (**c**) or YAP (**d**) in confluent HaCaT cells treated with 100 μM blebbistatin (Blebb) or DMSO (for control) for 6 h. *P < 0.001. *n* = 42 cells (in two independent experiments) for each bar. (**e** and **f**) Confluent HaCaT cells cultured for 40 h were treated for 6 h with 100 μM blebbistatin (Blebb) or DMSO (for control) together with or without 75 μM iCRT3 (**e**) or 2 μM Verteporfin (VP) (**f**). The cells were then incubated with EdU for 2 h in the presence of the same combination of the drugs. The percentages of EdU positive cells are shown. *P < 0.005; **P < 0.001; N.S., no significant difference. *n* = 8 (>50 cells each) for each bar. (**g**) Effects of cell density and actomyosin activity on YAP phosphorylation. HaCaT cells cultured for 40 h under the sparse and confluent conditions were treated with 100 μM blebbistatin (Blebb) or DMSO (for control) for 6 h, and then lysed and immunoblotted for Ser127-phosphorylated YAP (pYAP), YAP, β-catenin and actin. Similar results were obtained in two independent experiments.

β-Catenin and YAP play significant roles in the regulation of keratinocyte proliferation^35^,^39^, and downregulations of nuclear localization of and transcriptional activation by these proteins are reportedly involved in contact inhibition of cell proliferation^17^,^40^,^41^. We therefore asked whether actomyosin-dependent inhibition of proliferation of confluent keratinocytes is mediated by downregulation of β-catenin and YAP signaling. In confluent HaCaT cells, β-catenin clearly localized at AJs (in particular at apical AJs; see Fig. 2a), and YAP was distributed throughout the cytoplasm and the nucleus (Fig. 5a,b). However, myosin II inhibition with blebbistatin caused nuclear accumulation of these proteins (Fig. 5a–d), indicating that actomyosin activity sequesters β-catenin and YAP from their nuclear accumulation. Depletion of α-catenin expression also increased nuclear accumulation of β-catenin and YAP (Fig. 2b and Supplementary Fig. S6), which suggests involvement of the actin-AJ connection in retaining β-catenin and YAP in the cytoplasm.

We then examined whether actomyosin activity inhibits β-catenin- and YAP-driven proliferation of keratinocytes. When the transcription complex formation of β-catenin with Tcf was inhibited with iCRT3^42^, the promoting effect of myosin II inhibition on proliferation of confluent cells was eliminated (Fig. 5e). Inhibition of the YAP-TEAD transcription complex formation by the Verteporfin treatment^43^ (Fig. 5f) or shRNA-mediated depletion of YAP1 expression (Supplementary Fig. S7a,b) also abrogated the myosin II inhibition-induced increase in cell proliferation. These results indicate that although transcriptional activation by β-catenin and YAP promotes proliferation of confluent keratinocytes when actomyosin activity is low, elevation of actomyosin activity attenuates the β-catenin- and YAP-dependent proliferation. Surprisingly, depletion of β-catenin expression markedly increased proliferation of confluent keratinocytes (Supplementary Fig. S7a,b). We also found that β-catenin depletion in confluent keratinocytes caused actomyosin-independent accumulation of YAP in the nucleus (Supplementary Fig. S7c). These results reveal two opposing effects of β-catenin on proliferation of confluent keratinocytes; a promoting effect depending on β-catenin-mediated transcriptional activation and an inhibitory effect probably mediated by β-catenin-dependent attenuation of YAP nuclear accumulation.

It is not clear at present how actomyosin activity in confluent keratinocytes attenuates nuclear accumulation of β-catenin and YAP. However, development of AJs in response to actomyosin-generated tensile force acting on E-cadherin-mediated adhesion complexes^32^ might sequester β-catenin at these sites. This idea is supported by our observations that disassembly of β-catenin-rich apical AJs upon myosin II inhibition (by blebbistatin treatment) or upon ablation of the E-cadherin-actin connection (by depletion of α-catenin expression) increased nuclear accumulation of β-catenin (Fig. 5c and Supplementary Fig. S6b). Actomyosin-dependent retention of β-catenin in the cytoplasm may facilitate formation of the cytoplasmic complex of β-catenin and YAP and thereby hamper nuclear accumulation of YAP^44^, which is consistent with our result that β-catenin depletion induced nuclear accumulation of YAP.

Phosphorylation of YAP by the kinase cascade of Hippo signaling is another major mechanism for regulating nuclear accumulation of YAP; Hippo pathway-mediated Ser127 phosphorylation of YAP causes exclusion of YAP from the nucleus^45^. HaCaT keratinocytes with normal actomyosin activity showed a confluence-dependent decrease in nuclear accumulation of YAP (Supplementary Fig. S8a), which was associated with a confluence-dependent increase in YAP phosphorylation (Fig. 5g). This implies that HaCaT cells retain the Hippo-mediated mechanism for the regulation of YAP nuclear localization. By contrast, actomyosin inhibition-induced nuclear accumulation of YAP in confluent HaCaT cells was not associated with a change in YAP phosphorylation (Fig. 5g), suggesting that actomyosin-dependent regulation of nuclear accumulation of YAP in these cells is mediated by a mechanism distinct from Hippo signaling. Hippo-independent regulation of YAP nuclear accumulation has been reported also in other cell types, in particular in terms of mechanical regulation of YAP localization^17^,^46^.

While E-cadherin-mediated cell-cell contacts without actomyosin activity were found to promote keratinocyte proliferation (Fig. 1), the extent of YAP nuclear accumulation under actomyosin inhibition was lower in confluent keratinocytes than in sparse ones (Supplementary Fig. S8a). This suggests that the E-cadherin ligation-induced increase in proliferation of actomyosin-inhibited keratinocytes is not mediated by increased YAP accumulation in the nucleus. Alternatively, it has been reported that E-cadherin ligation stimulates cell proliferation through p120-catenin and Rac1 at a sub-confluent cell density^47^. Involvement of Rac1 in ERK-dependent epithelial cell proliferation has also been suggested^48^. Therefore, the p120-catenin-Rac1-ERK pathway may be involved in the E-cadherin-dependent increase in proliferation of actomyosin-inhibited keratinocytes, which should be tested in future studies.

Recent studies have demonstrated that proliferation of confluent epithelial cells of non-keratinocyte types is increased in response to mechanical stretch of the underlying extracellular substrates^17^,^19^. In principle, stretch of extracellular substrates increases tensile force at both AJs and cell-substrate adhesions called focal adhesions (FAs). Since tensile force at FAs promotes cell proliferation mainly via activation of the FAK-MAP kinase pathway^49^, the stretch-induced increase in epithelial cell proliferation may be mediated by the pro-proliferative effect of tensile force at FAs. By contrast, while actomyosin activity also potentially increases tensile forces at AJs and FAs^34^,^50^, proliferation of confluent keratinocytes was reduced by actomyosin activity (this study). It is noteworthy that FA formation was largely suppressed in confluent keratinocytes (Supplementary Fig. S8b), which may make the pro-proliferative effect of actomyosin-based tensile force at FAs minimal. Instead, the anti-proliferative effect of actomyosin force at AJs may become dominant in these cells. Consistent with this idea, proliferation of sparse keratinocytes, which possessed well-developed FAs (Supplementary Fig. S8b), was increased by actomyosin activity (Fig. 1b).

Architectural and mechanical characteristics of apical AJs were apparently different between keratinocytes and non-keratinocyte type epithelial cells. Although apical AJs in HaCaT keratinocytes were punctate, actomyosin-dependent and connected to radial actin cables, those in MCF-10A epithelial cells were continuous, actomyosin-independent and associated with the mesh-like structure of F-actin (Supplementary Fig. S4). Keratinocytes form punctate AJs and connected actin cables when they are stratifying, which potentially contributes to promoting keratinocyte stratification^51^. Therefore, while our results in this study suggest that tensile stress developed at the apical AJ-actin cable complexes inhibits proliferation of confluent keratinocytes, this inhibitory mechanism of cell proliferation may be specialized for keratinocytes at the stratification stage. On the other hand, the actomyosin insensitivity of AJs in non-keratinocyte epithelial cells (Supplementary Fig. S4) might underpin actomyosin-independent contact inhibition in these cells (Supplementary Fig. S2d).

Keratinocytes should have a mechanosensor molecule(s) that senses tensile stress at the apical AJ-actin cable complexes and transduces it into inhibition of β-catenin- and YAP-dependent cell proliferation. α-Catenin works as a potential mechanosensor molecule at AJs^33^,^52^; however, the mechanosensor responsible for inducing contact inhibition in keratinocytes is unknown and should be identified in future studies. While loss of contact inhibition in cancer cells leads to their overgrowth and tumorigenesis^8^,^9^, exogenetic activation of the to-be-identified mechanosensor in transformed keratinocytes may provide a novel therapeutic approach for prohibiting tumorigenesis in keratinocyte carcinomas.

## Methods

### Cell culture

Human HaCaT keratinocytes (Cell Lines Service, Eppelheim, Germany) and HEK293T cells were maintained in high-glucose Dulbecco’s modified Eagle’s medium (Life Technologies, Carlsbad, CA) supplemented with 10% fetal bovine serum (Life Technologies). MCF-10A human mammary gland epithelial cells were maintained in HuMEC medium (Life Technologies). For experiments, trypsinized HaCaT keratinocytes or MCF-10A epithelial cells were seeded at the cell densities of 0.3 × 10^4^ cells/cm^2^ (sparse) or 10 × 10^4^ cells/cm^2^ (confluent) onto glass bottom dishes or cell culture plastic plates precoated with 50 μg/ml collagen (Koken, Tokyo), and cultured for 40 h unless otherwise indicated. When cells were seeded at the confluent cell density (i.e., 10 × 10^4^ cells/cm^2^), surfaces of the glass bottom dishes/cell culture plastic plates were totally covered with cells. In sparse cultures, cells forming clusters were excluded from analyses.

### Antibodies and chemicals

The rabbit polyclonal antibody (pAb) against β-catenin (ab6302) and the mouse monoclonal antibody (mAb) against DSG3 (3G133) were purchased from Abcam (Cambridge, UK). The mouse inhibitory mAb against E-cadherin (67A4) and the mouse mAb against cyclin E (HE12) were from Merck Millipore (Billerica, MA). The rabbit pAb against Ser127-phosphorylated YAP (#4911) and the rabbit mAb against E-cadherin (24E10) were from Cell Signaling Technology (Danvers, MA). The rabbit pAb against YAP1 (NB110-58358) was from Novus Biologicals (Littleton, CO). The mouse mAbs against β-actin (AC-15) and vinculin (hVIN-1), and the rabbit pAb against α-catenin (C2081) were from Sigma Chemical (St. Louis, MO). Mouse IgG1 for the isotype control (2E12) was from Medical & Biological Laboratories (Nagoya, Japan). Alexa Fluor 488-goat anti-rabbit IgG, Alexa Fluor 488-goat anti-mouse IgG and Alexa Fluor 546-goat anti-mouse IgG antibodies, and Alexa Fluor 546-phalloidin were from Life Technologies. Horseradish peroxidase-conjugated anti-mouse IgG and anti-rabbit IgG antibodies were from GE Healthcare (Little Chalfont, UK) and Life Technologies, respectively. The recombinant E-cadherin-Fc chimeric protein, Y-27632, iCRT3 and Verteporfin were from Sigma Chemical. Blebbistatin was from Toronto Research Chemicals (North York, Canada). The RhoA activator CN03 was from Cytoskeleton (Denver, CO).

### EdU incorporation

Cells were treated with drugs/compounds for 6 h when indicated and further incubated for 2 h with 10 μM EdU in the presence of the same set of the drugs/compounds. These cells were fixed and permeabilized with 4% formaldehyde and 0.5% Triton X-100, respectively, in PBS, and incorporated EdU was visualized with Alexa Fluor 488-azide using the Click-iT technology (Life Technologies). Total nuclei were stained with 5 μg/ml Hoechst 33342 (Life Technologies). While blebbistatin treatment caused incomplete cell division as reported previously^29^ (Supplementary Fig. S1c), 6-h treatment of blebbistatin used in this study did not significantly alter the ratio of bi-nucleate cells (Supplementary Fig. S9a,b), and bi-nucleate cells were excluded from analyses of the EdU incorporation assay. Longer time treatment (>15 h) with blebbistatin caused significant cell death probably due to its cytotoxic effect (Supplementary Fig. S9c and Supplementary Video 3).

### Immunofluorescence

Cells cultured for 40 h and then treated with drugs/compounds for 6 h when indicated were fixed and permeabilized for 30 min with 4% formaldehyde and 0.2% Triton X-100 in the cytoskeleton stabilizing buffer (137 mM NaCl, 5 mM KCl, 1.1 mM Na_2_HPO_4_, 0.4 mM KH_2_PO_4_, 4 mM NaHCO_3_, 2 mM MgCl_2_, 5.5 mM glucose, 2 mM EGTA, and 5 mM PIPES, pH 6.1). This was followed by blocking with 1% BSA in the cytoskeleton stabilizing buffer for 30 min. The cells were then incubated with primary antibodies for 40 min, washed, and further incubated with secondary antibodies (and fluorescent phalloidin, when necessary) for 40 min. Antibodies were diluted to 1:200 in cytoskeleton stabilizing buffer containing 1% BSA. All immunofluorescence experiments were repeated at least twice, and typical images are shown in figures.

### shRNA-mediated depletion of α-catenin, β-catenin and YAP1

To generate retroviruses expressing shRNAs against α-catenin, β-catenin and YAP1, the target sequences of 5′-GACTTAGGAATCCAGTATA-3′ (for human catenin alpha-1), 5′-CTATCAAGATGATGCAGAA-3′ (for human catenin beta-1) and 5′-GACATCTTCTGGTCAGAGA-3′ (for human YAP1) were inserted into the pSUPER.retro.puro retroviral vector. For control, the non-targeting sequence 5′-ATAGTCACAGACATTAGGT-3′ was introduced. The shRNA-containing vector was co-transfected with the pE-ampho vector into HEK293T cells using the GeneJuice transfection reagent (Merck Millipore). Supernatants containing viral particles were collected 48 h after the transfection, filtered through 0.45-μm syringe filters, and used for infection into HaCaT cells in the presence of 8 μg/ml Polybrene (Sigma Chemical). Infected HaCaT cells were selected with 1.5 μg/ml puromycin (Sigma Chemical).

### Immunoblot

Cells were lysed with 2× lithium dodecyl sulfate sample buffer (Life Technologies) containing 2.5% β-mercaptoethanol. The lysate samples were resolved by SDS-PAGE (4–12% Bis-Tris gel; Life Technologies), transferred onto a polyvinylidene fluoride membrane (Merck Millipore), and probed with antibodies. Immuno-reactive bands were detected with Chemi-Lumi One Super (Nacalai Tesque, Kyoto, Japan). All immunoblot experiments were repeated at least twice.

### Magnetic bead assay

The recombinant E-cadherin-Fc chimeric protein and the anti-DSG3 antibody were covalently coupled to protein G-conjugated 3-μm magnetic beads (Bio-Rad, Hercules, CA) with 20 mM dimethyl pimelimidate•2 HCl (DMP) (Thermo Fisher Scientific, Rockford, IL), as described previously^53^. HaCaT cells were incubated for 1 h with the E-cadherin-Fc- or anti-DSG3 antibody-coupled beads that were suspended in DMEM containing 100 μM blebbistatin. After washing free beads out, magnetic force was applied for 6 h to cell-bound beads in the presence of 100 μM blebbistatin by placing a neodymium magnet with the surface magnetic flux density of 1250 gauss onto the glass bottom dish. For the EdU incorporation assay, the cells were further incubated with 10 μM EdU for 2 h in the presence of 100 μM blebbistatin and the neodymium magnet.

### Microscope image acquisition

For fluorescence imaging, cells were observed using an epi-fluorescence inverted microscope (ECLIPSE TE2000-U, Nikon, Tokyo) equipped with an air (NA 0.30, 10 x; Plan Fluor, Nikon) or an oil immersion (NA 1.45, 100 x; Plan Apo TIRF, Nikon) objective and a complementary metal oxide semiconductor camera (ORCA-Flash4.0 C11440-22CU, Hamamatsu Photonics, Hamamatsu, Japan). The Metamorph software (version 7.8, Molecular Devices, Sunnyvale, CA) was used for image acquisition. Acquired images were analyzed offline using the public domain software ImageJ (version 1.45 f). For quantification of fluorescence intensities in the cytoplasm, the region of cell-cell junctions was excluded from analyses, because it was difficult to determine to which neighboring cells individual pixels in the cell-cell junction region were attributed.

For time-lapse imaging of live cells, cells were observed at 37 °C and 100% humidity in 5% CO_2_ using an inverted microscope (BZ-X710, Keyence, Osaka, Japan) equipped with a 20× air objective (NA 0.45; S Plan Fluor ELWD, Nikon). Confluent HaCaT keratinocytes cultured for 40 h after seeding the cells were mounted on the microscope stage, and blebbistatin (the final concentration of 100 μM) or DMSO (control) was added to the cells. After a 6-h incubation, time-lapse imaging was conducted in the presence of blebbistatin or DMSO. Phase contrast images were captured at every 5 min for 35 h. Far-red light was used for illumination to reduce phototoxicity against cells and deactivation of blebbistatin.

### Statistical analysis

Bar graphs were presented as means ± SD. In the EdU incorporation assay, eight image fields in two independent experiments (>50 cells in each field) were analyzed for each condition. Statistical significance was assessed using Student’s two-tailed, unpaired *t*-test.

## Additional Information

**How to cite this article:** Hirata, H. *et al*. Actomyosin contractility provokes contact inhibition in E-cadherin-ligated keratinocytes. *Sci. Rep.* **7**, 46326; doi: 10.1038/srep46326 (2017).

**Publisher's note:** Springer Nature remains neutral with regard to jurisdictional claims in published maps and institutional affiliations.

## Supplementary Material

Supplementary Information

Supplementary Video 1

Supplementary Video 2

Supplementary Video 3

## References

[b1] Conlon I. & Raff M. Size control in animal development. Cell 96, 235–244 (1999). 9988218 10.1016/s0092-8674(00)80563-2

[b2] Macara I. G., Guyer R., Richardson G., Huo Y. & Ahmed S. M. Epithelial homeostasis. Curr. Biol. 24, R815–R825 (2014). 25202877 10.1016/j.cub.2014.06.068 PMC4196707

[b3] Barker N., Bartfeld S. & Clevers H. Tissue-resident adult stem cell populations of rapidly self-renewing organs. Cell Stem Cell 7, 656–670 (2010). 21112561 10.1016/j.stem.2010.11.016

[b4] Doupé D. P. . A single progenitor population switches behavior to maintain and repair esophageal epithelium. Science 337, 1091–1093 (2012). 22821983 10.1126/science.1218835 PMC3527005

[b5] de la Cova C., Abril M., Bellosta P., Gallant P. & Johnston L. A. Drosophila Myc regulates organ size by inducing cell competition. Cell 117, 107–116 (2004). 15066286 10.1016/s0092-8674(04)00214-4

[b6] Vincent J. P., Fletcher A. G. & Baena-Lopez L. A. Mechanisms and mechanics of cell competition in epithelia. Nat. Rev. Mol. Cell Biol. 14, 581–591 (2013). 23942450 10.1038/nrm3639

[b7] Eisenhoffer G. T. . Crowding induces live cell extrusion to maintain homeostatic cell numbers in epithelia. Nature 484, 546–549 (2012). 22504183 10.1038/nature10999 PMC4593481

[b8] McClatchey A. I. & Yap A. S. Contact inhibition (of proliferation) redux. Curr. Opin. Cell Biol. 24, 685–694 (2012). 22835462 10.1016/j.ceb.2012.06.009

[b9] Hanahan D. & Weinberg R. A. Hallmarks of cancer: the next generation. Cell 144, 646–674 (2011). 21376230 10.1016/j.cell.2011.02.013

[b10] St Croix B. . E-cadherin-dependent growth suppression is mediated by the cyclin-dependent kinase inhibitor p27^KIP1^. J. Cell Biol. 142, 557–571 (1998). 9679152 10.1083/jcb.142.2.557 PMC2133056

[b11] Motti M. L. . Reduced E-cadherin expression contributes to the loss of p27^kip1^-mediated mechanism of contact inhibition in thyroid anaplastic carcinomas. Carcinogenesis 26, 1021–1034 (2005). 15718252 10.1093/carcin/bgi050

[b12] Perrais M., Chen X., Perez-Moreno M. & Gumbiner B. M. E-cadherin homophilic ligation inhibits cell growth and epidermal growth factor receptor signaling independently of other cell interactions. Mol. Biol. Cell 18, 2013–2025 (2007). 17392517 10.1091/mbc.E06-04-0348 PMC1877107

[b13] Kim N. G., Koh E., Chen X. & Gumbiner B. M. E-cadherin mediates contact inhibition of proliferation through Hippo signaling-pathway components. Proc. Natl. Acad. Sci. USA 108, 11930–11935 (2011). 21730131 10.1073/pnas.1103345108 PMC3141988

[b14] Sun C. C., Chiu H. T., Lin Y. F., Lee K. Y. & Pang J. H. S. Y-27632, a ROCK inhibitor, promoted limbal epithelial cell proliferation and corneal wound healing. PLoS One 10, e0144571 (2015). 26673160 10.1371/journal.pone.0144571 PMC4684472

[b15] Roshan A. . Human keratinocytes have two interconvertible modes of proliferation. Nat. Cell Biol. 18, 145–156 (2016). 26641719 10.1038/ncb3282 PMC4872834

[b16] Puliafito A. . Collective and single cell behavior in epithelial contact inhibition. Proc. Natl. Acad. Sci. USA 109, 739–744 (2012). 22228306 10.1073/pnas.1007809109 PMC3271933

[b17] Aragona M. . A mechanical checkpoint controls multicellular growth through YAP/TAZ regulation by actin-processing factors. Cell 154, 1047–1059 (2013). 23954413 10.1016/j.cell.2013.07.042

[b18] Kim J. H. & Asthagiri A. R. Matrix stiffening sensitizes epithelial cells to EGF and enables the loss of contact inhibition of prolliferation. J. Cell Sci. 124, 1280–1287 (2011). 21429934 10.1242/jcs.078394 PMC3065384

[b19] Benham-Pyle B., Pruitt B. L. & Nelson W. J. Mechanical strain induces E-cadherin-dependent Yap1 and β-catenin activation to drive cell cycle entry. Science 348, 1024–1027 (2015). 26023140 10.1126/science.aaa4559 PMC4572847

[b20] Chaudhuri P. K., Pan C. Q., Low B. C. & Lim C. T. Topography induces differential sensitivity on cancer cell proliferation via Rho-ROCK-Myosin contractility. Sci. Rep. 6, 19672 (2016). 26795068 10.1038/srep19672 PMC4726280

[b21] Hufnagel L., Teleman A. A., Rouault H., Cohen S. M. & Shraiman B. I. On the mechanism of wing size determination in fly development. Proc. Natl. Acad. Sci. USA 104, 3835–3840 (2007). 17360439 10.1073/pnas.0607134104 PMC1820670

[b22] Simpson C. L., Patel D. M. & Green K. J. Deconstructing the skin: cytoarchitectural determinants of epidermal morphogenesis. Nat. Rev. Mol. Cell Biol. 12, 565–580 (2011). 21860392 10.1038/nrm3175 PMC3280198

[b23] Vasioukhin V., Bauer C., Degenstein L., Wise B. & Fuchs E. Hyperproliferation and defects in epithelial polarity upon conditional ablation of α-catenin in skin. Cell 104, 605–617 (2001). 11239416 10.1016/s0092-8674(01)00246-x

[b24] Dietrich C., Scherwat J., Faust D. & Oesch F. Subcellular localization of β-catenin is regulated by cell density. Biochem. Biophys. Res. Commun. 292, 195–199 (2002). 11890692 10.1006/bbrc.2002.6625

[b25] Regl G. . The zinc-finger transcription factor GLI2 antagonizes contact inhibition and differentiation of human epidermal cells. Oncogene 23, 1263–1274 (2004). 14691458 10.1038/sj.onc.1207240

[b26] Huang J., Zhang J., Pathak A., Li J. & Stouffer G. A. Perivascular delivery of blebbistatin reduces neointimal hyperplasia after carotid injury in the mouse. J. Pharmacol. Exp. Ther. 336, 116–126 (2011). 20956482 10.1124/jpet.110.174615 PMC3014304

[b27] Chiu H. C., Chang T. Y., Huang C. T., Chao Y. S. & Hsu J. T. A. EGFR and myosin II inhibitors cooperate to suppress EGFR-T790M-mutant NSCLC cells. Mol. Oncol. 6, 299–310 (2012). 22366308 10.1016/j.molonc.2012.02.001 PMC5528336

[b28] Mih J. D., Marinkovic A., Liu F., Sharif A. S. & Tschumperlin D. J. Matrix stiffness reverses the effect of actomyosin tension on cell proliferation. J. Cell Sci. 125, 5974–5983 (2012). 23097048 10.1242/jcs.108886 PMC3585515

[b29] Straight A. F., Cheung A., Limouze J., Chen I., Westwood N. J., Sellers J. R. & Mitchison T. J. Dissecting temporal and spatial control of cytokinesis with a myosin II inhibitor. Science 299, 1743–1747 (2003). 12637748 10.1126/science.1081412

[b30] Tinkle C. L., Lechler T., Pasolli H. A. & Fuchs E. Conditional targeting of E-cadherin in skin: insights into hyperproliferative and degenerative responses. Proc. Natl. Acad. Sci. USA 101, 552–557 (2004). 14704278 10.1073/pnas.0307437100 PMC327185

[b31] Kovacs E. M., Ali R. G., McCormack A. J. & Yap A. S. E-cadherin homophilic ligation directly signals through Rac and phosphatidylinositol 3- kinase to regulate adhesive contacts. J. Biol. Chem. 277, 6708–6718 (2002). 11744701 10.1074/jbc.M109640200

[b32] Liu Z. . Mechanical tugging force regulates the size of cell-cell junctions. Proc. Natl. Acad. Sci. USA. 107, 9944–9949 (2010). 20463286 10.1073/pnas.0914547107 PMC2890446

[b33] Yonemura S., Wada Y., Watanabe T., Nagafuchi A. & Shibata M. α-Catenin as a tension transducer that induces adherens junction development. Nat. Cell Biol. 12, 533–542 (2010). 20453849 10.1038/ncb2055

[b34] Borghi N. . E-cadherin is under constitutive actomyosin-generated tension that is increased at cell-cell contacts upon externally applied stretch. Proc. Natl. Acad. Sci. USA 109, 12568–12573 (2012). 22802638 10.1073/pnas.1204390109 PMC3411997

[b35] Schlegelmilch K. . Yap1 acts downstream of α-catenin to control epidermal proliferation. Cell 144, 782–795 (2011). 21376238 10.1016/j.cell.2011.02.031 PMC3237196

[b36] Silvis M. R. . α-Catenin is a tumor suppressor that controls cell accumulation by regulating the localization and activity of the transcriptional coactivator Yap1. Sci. Signal. 4, ra33 (2011). 21610251 10.1126/scisignal.2001823 PMC3366274

[b37] Schmidt G. . Gln 63 of Rho is deamidated by Escherichia coli cytotoxic necrotizing factor-1. Nature 387, 725–729 (1997). 9192900 10.1038/42735

[b38] Flatau G. . Toxin-induced activation of the G protein p21 Rho by deamidation of glutamine. Nature 387, 729–733 (1997). 9192901 10.1038/42743

[b39] Samuel M. S. . Actomyosin-mediated cellular tension drives increased tissue stiffness and β-catenin activation to induce epidermal hyperplasia and tumor growth. Cancer Cell 19, 776–791 (2011). 21665151 10.1016/j.ccr.2011.05.008 PMC3115541

[b40] Zhao B. . Inactivation of YAP oncoprotein by the Hippo pathway is involved in cell contact inhibition and tissue growth control. Genes Dev. 21, 2747–2761 (2007). 17974916 10.1101/gad.1602907 PMC2045129

[b41] Ose R., Yanagawa T., Ikeda S., Ohara O. & Koga H. PCDH24-induced contact inhibition involves downregulation of β-catenin signaling. Mol. Oncol. 3, 54–66 (2009). 19383367 10.1016/j.molonc.2008.10.005 PMC5527873

[b42] Gonsalves F. C. . An RNAi-based chemical genetic screen identifies three small-molecule inhibitors of the Wnt/*wingless* signaling pathway. Proc. Natl. Acad. Sci. USA 108, 5954–5963 (2011). 21393571 10.1073/pnas.1017496108 PMC3076864

[b43] Liu-Chittenden Y. . Genetic and pharmacological disruption of the TEAD-YAP complex suppresses the oncogenic activity of YAP. Genes Dev. 26, 1300–1305 (2012). 22677547 10.1101/gad.192856.112 PMC3387657

[b44] Azzolin L. . YAP/TAZ incorporation in the β-catenin destruction complex orchestrates the Wnt response. Cell 158, 157–170 (2014). 24976009 10.1016/j.cell.2014.06.013

[b45] Gumbiner B. M. & Kim N. G. The Hippo-YAP signaling pathway and contact inhibition of growth. J. Cell Sci. 127, 709–717 (2014). 24532814 10.1242/jcs.140103 PMC3924201

[b46] Dupont S. . Role of YAP/TAZ in mechanotransduction. Nature 474, 179–183 (2011). 21654799 10.1038/nature10137

[b47] Liu W. F., Nelson C. M., Pirone D. M. & Chen C. S. E-cadherin engagement stimulates proliferation via Rac1. J. Cell Biol. 173, 431–441 (2006). 16682529 10.1083/jcb.200510087 PMC2063843

[b48] Aoki K., Kumagai Y., Sakurai A., Komatsu N., Fujita Y., Shionyu C. & Matsuda M. Stochastic ERK activation induced by noise and cell-to-cell propagation regulates cell density-dependent proliferation. Mol. Cell 52, 529–540 (2013). 24140422 10.1016/j.molcel.2013.09.015

[b49] Provenzano P. P. & Keely P. J. Mechanical signaling through the cytoskeleton regulates cell proliferation by coordinated focal adhesion and Rho GTPase signaling. J. Cell Sci. 124, 1195–1205 (2011). 21444750 10.1242/jcs.067009 PMC3065381

[b50] Tan J. L., Tien J., Pirone D. M., Gray D. S., Bhadriraju K. & Chen C. S. Cells lying on a bed of microneedles: an approach to isolate mechanical force. Proc. Natl. Acad. Sci. USA 100, 1484–1489 (2003). 12552122 10.1073/pnas.0235407100 PMC149857

[b51] Vaezi A., Bauer C., Vasioukhin V. & Fuchs E. Actin cable dynamics and Rho/Rock orchestrate a polarized cytoskeletal architecture in the early steps of assembling a stratified epithelium. Dev. Cell 3, 367–381 (2002). 12361600 10.1016/s1534-5807(02)00259-9

[b52] Yao M. . Force-dependent conformational switch of α-catenin controls vinculin binding. Nat. Commun. 5, 4525 (2014). 25077739 10.1038/ncomms5525

[b53] Hirata H., Tatsumi H., Lim C. T. & Sokabe M. Force-dependent vinculin binding to talin in live cells: a crucial step in anchoring the actin cytoskeleton to focal adhesions. Am. J. Physiol. Cell Physiol. 306, C607–C620 (2014). 24452377 10.1152/ajpcell.00122.2013

