# Supplementary material for: Actomyosin contractility provokes contact inhibition in E-cadherin-ligated keratinocytes
Source: Sci Rep. 2017 Apr 13;7:46326. doi: 10.1038/srep46326 (PMC5390311; doi:10.1038/srep46326)
Supplement: Supplementary Information [file srep46326-s1.pdf]

Actomyosin contractility provokes contact inhibition in E-cadherin-ligated keratinocytes

Hiroaki Hirata, Mikhail Samsonov and Masahiro Sokabe

Supplementary Information

Supplementary Figure Legends

Supplementary Figure S1. Effects of inhibition of Rho kinase and myosin II on proliferation and cytokinesis in confluent keratinocytes. (**a**) Confluent HaCaT cells cultured for 40 h were incubated with EdU for 2 h and then stained for nuclei (Hoechst) and cyclin E. Scale bar, 50 μm. (**b**) Confluent HaCaT cells cultured for 40 h were treated with 60 μM Y-27632 or DMSO (for control) for 6 h, and then incubated with EdU for 2 h in the presence of Y-27632 or DMSO. The percentages of EdU positive cells are shown. *, $P < 0.01$. $n = 8$ (> 50 cells each) for each bar. (**c**) Time-lapse images of cytokinetic events in confluent HaCaT cells in the presence of either 100 μM blebbistatin (Blebb) or DMSO (for control). Yellow arrows indicate the cells undergoing cytokinetic events. Note that the blebbistatin-treated cell failed to divide. Scale bar, 30 μm. (**d**) Frequencies of cytokinetic events (including incomplete cell division) in confluent HaCaT cells in the presence of 100 μM blebbistatin (Blebb) or DMSO (for control). Cell behaviors in the first 5 h of the time-lapse imaging were analyzed (see Methods). *, $P < 0.05$. $n = 3$ (> 130 cells in each) for each bar.

Supplementary Figure S2. Time courses of progression of contact inhibition in HaCaT and MCF-10A cells. (**a** and **c**) HaCaT (**a**) and MCF-10A (**c**) cells cultured for the indicated time after seeding the cells under the sparse and confluent conditions were incubated with EdU for 2 h. The percentages of EdU positive cells are shown. Each

point represents the mean ± SD for n = 8 (> 50 cells each). (**b**) Confluent HaCaT cells cultured for 64 h after seeding the cells were treated with 100 μM blebbistatin (Blebb) or DMSO (for control) for 6 h, and then incubated with EdU for 2 h in the presence of blebbistatin or DMSO. The percentages of EdU positive cells are shown. *, $P < 0.05$. n = 8 (> 50 cells each) for each bar. (**d**) MCF-10A cells cultured for 40 h under the sparse and confluent conditions were treated with 100 μM blebbistatin (Blebb) or DMSO (for control) for 6 h, and then incubated with EdU for 2 h in the presence of blebbistatin or DMSO. The percentages of EdU positive cells are shown. N.S., no significant difference. n = 8 (> 50 cells each) for each bar.

Supplementary Figure S3. E-cadherin dependency of adherens junctions in confluent keratinocytes. (**a**) Confluent HaCaT cells cultured for 40 h were treated with 100 μM blebbistatin (Blebb) or DMSO (for control) for 6 h, and then stained for E-cadherin and F-actin. Apical, middle and basal focal planes of the cells are shown. A magnified and merged image of the boxed region (DMSO, apical) is also shown. Scale bars, 5 μm for the magnified and merged image, and 10 μm for others. (**b**) Confluent HaCaT cells cultured for 40 h were treated with 5 μg/ml anti-E-cadherin inhibitory antibody (anti-Ecad) or control IgG for 6 h, and then stained for β-catenin and F-actin. Apical focal planes of the cells are shown. A magnified and merged image of the boxed region (IgG) is also shown. Scale bars, 5 μm for the magnified and merged image, and 20 μm for others.

Supplementary Figure S4. Actomyosin dependency of adherens junctions in confluent HaCaT and MCF-10A cells. HaCaT and MCF-10A cells cultured for 40 h

under the confluent condition were treated with 100 μM blebbistatin (Blebb) or DMSO (for control) for 6 h, and then stained for β-catenin and F-actin. Apical, middle and basal focal planes of the cells are shown. Magnified and merged images of the boxed regions (DMSO, apical) are also shown. Scale bars, 10 μm for the magnified and merged images, and 20 μm for others.

Supplementary Figure S5. Effects of enforced activation of RhoA on adherens junctions and cell proliferation in confluent MCF-10A cells. (**a** and **b**) Confluent MCF-10A cells cultured for 40 h were treated with or without 5 μg/ml CN03 for 6 h and then subjected to either immunostaining for β-catenin and F-actin (**a**) or the EdU incorporation assay (**b**). Apical, middle and basal focal planes of the cells are shown in (**a**). Scale bars, 20 μm. The percentages of EdU positive cells are shown in (**b**). N.S., no significant difference. $n = 8$ (> 50 cells each) for each bar.

Supplementary Figure S6. α-Catenin depletion increases nuclear accumulation of β-catenin and YAP. (**a**) HaCaT cells expressing shRNA against α-catenin (shαCat) or non-targeting shRNA (shContl) were cultured for 40 h under the confluent condition, and then stained for YAP, nuclei (Hoechst) and F-actin. Scale bar, 20 μm. (**b** and **c**) The nuclear/cytoplasmic ratio of fluorescence intensities of β-catenin (**b**) or YAP (**c**) in confluent HaCaT cells expressing shRNA against α-catenin (shαCat) or non-targeting shRNA (shContl). *, $P < 0.001$. $n = 20$ cells (in two independent experiments) for each bar.

Supplementary Figure S7. β-Catenin depletion increases YAP nuclear

accumulation and cell proliferation in confluent keratinocytes. (**a**) After culturing for 40 h, confluent HaCaT cells expressing either shRNA against β-catenin (shβCat), shRNA against YAP1 (shYAP1) or non-targeting shRNA (shContl) were lysed and immunoblotted for YAP, β-catenin and actin. (**b**) After culturing for 40 h, confluent HaCaT cells expressing either shRNA against β-catenin (shβCat), shRNA against YAP1 (shYAP1) or non-targeting shRNA (shContl) were treated with 100 μM blebbistatin (Blebb) or DMSO (for control) for 6 h, and then incubated with EdU for 2 h in the presence of blebbistatin or DMSO. The percentages of EdU positive cells are shown. *, $P < 0.005$; N.S., no significant difference. $n = 8$ (> 50 cells each) for each bar. (**c**) Confluent HaCaT cells expressing shRNA against β-catenin (shβCat) or non-targeting shRNA (shContl) were cultured for 40 h, and then treated with 100 μM blebbistatin (Blebb) or DMSO (for control) for 6 h. These cells were stained for YAP, nuclei (Hoechst) and F-actin. Scale bars, 30 μm.

Supplementary Figure S8. Localization of YAP and vinculin in sparse and confluent keratinocytes. (**a**) Sparse and confluent HaCaT cells cultured for 40 h were treated with 100 μM blebbistatin (Blebb) or DMSO (for control) for 6 h, and then stained for YAP, nuclei (Hoechst) and F-actin. Scale bars, 50 μm. (**b**) Sparse and confluent HaCaT cells cultured for 40 h were stained for vinculin and F-actin. Scale bars, 20 μm.

Supplementary Figure S9. Effect of actomyosin inhibition on production of bi-nucleate cells. (**a**) Confluent HaCaT cells cultured for 40 h were treated with 100 μM blebbistatin (Blebb) or DMSO (for control) for 6 h, and then stained for nuclei

(Hoechst), β-catenin and F-actin. Bi-nucleate cells are indicated by arrows. Scale bar, 30 μm. (**b**) The percentages of bi-nucleate cells in confluent HaCaT cells treated with 100 μM blebbistatin (Blebb) or DMSO (for control) for 6 h. N.S., no significant difference. $n = 8$ (> 30 cells each) for each bar. (**c**) Time-lapse images of a HaCaT cell (yellow arrow) that underwent cell death after incomplete cell division in the presence of 100 μM blebbistatin (Blebb). Scale bar, 30 μm.

Supplementary Video 1. Time-lapse images of DMSO-treated HaCaT cells under the confluent condition. Confluent HaCaT cells cultured for 40 h after the cell seeding were pre-treated with DMSO for 6h, and then time-lapse imaging was conducted in the presence of DMSO. Phase contrast images captured at every 5 min for 35 h are shown at the speed of 25 frames per second.

Supplementary Video 2. Time-lapse images of blebbistatin-treated HaCaT cells under the confluent condition. Confluent HaCaT cells cultured for 40 h after the cell seeding were pre-treated with 100 μM blebbistatin for 6h, and then time-lapse imaging was conducted in the presence of blebbistatin. Phase contrast images captured at every 5 min for 35 h are shown at the speed of 25 frames per second.

Supplementary Video 3. Incomplete cell division followed by cell death in blebbistatin-treated HaCaT cells under the confluent condition. Confluent HaCaT cells cultured for 40 h after the cell seeding were pre-treated with 100 μM blebbistatin for 6h, and then time-lapse imaging was conducted in the presence of blebbistatin. Phase contrast images (captured at every 5 min for 13 h) of a cell undergoing

incomplete cell division followed by cell death are shown at the speed of 20 frames per second.

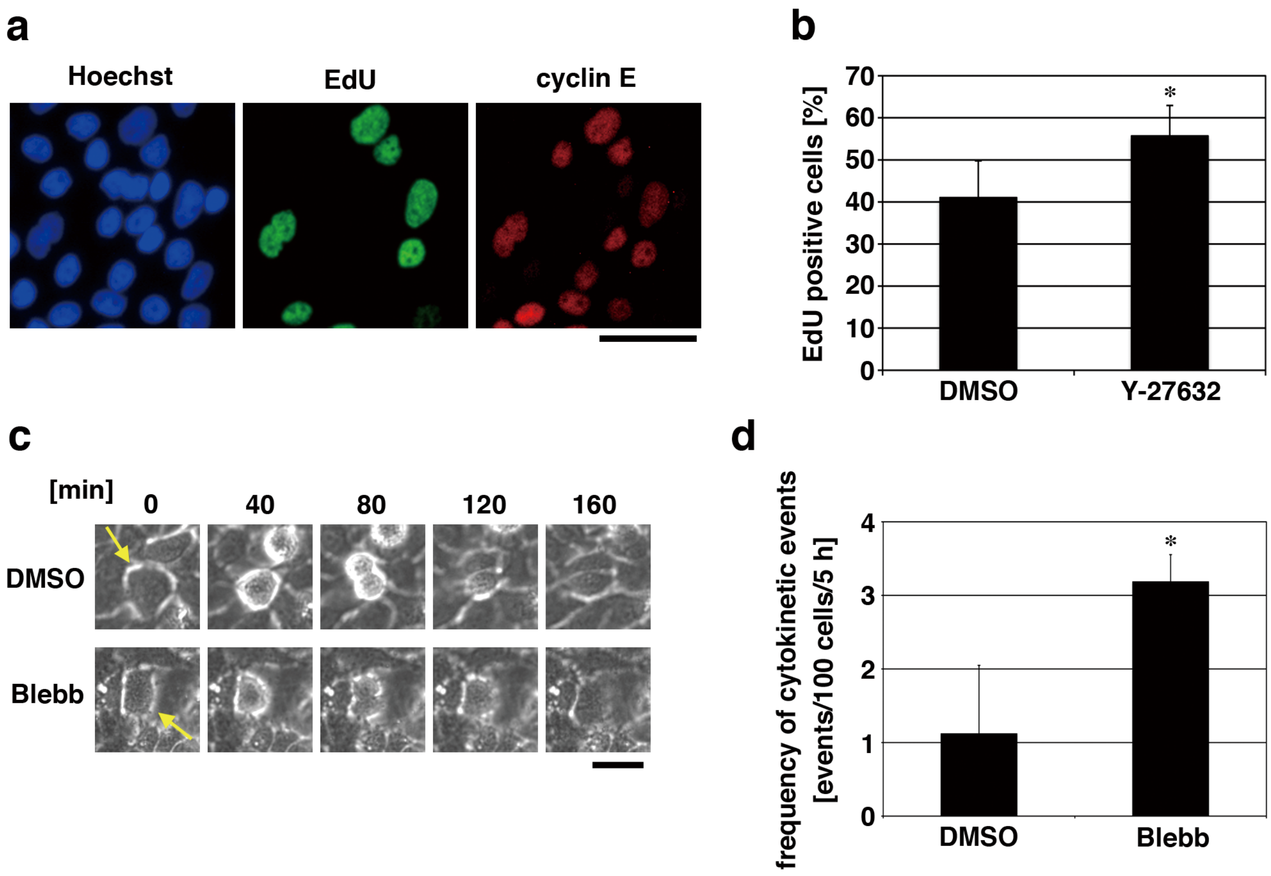

a
Hoechst
EdU
cyclin E
b
EdU positive cells [%]
70
60
50
40
30
20
10
0
*
DMSO
Y-27632
c
[min]
0
40
80
120
160
DMSO
Blebb
d
frequency of cytokinetic events
[events/100 cells/5 h]
4
3
2
1
0
*
DMSO
Blebb

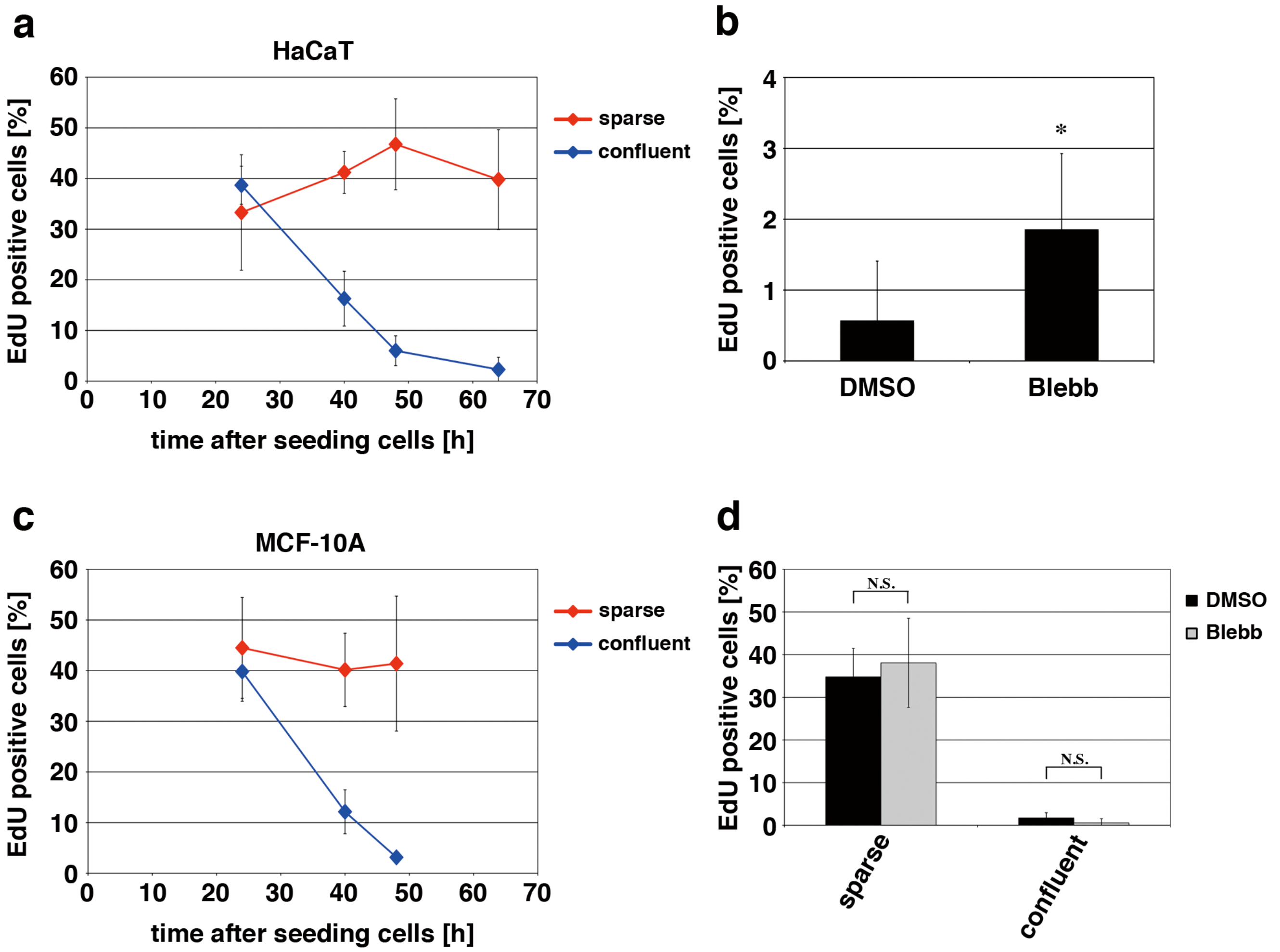
a
HaCaT
EdU positive cells [%]
time after seeding cells [h]
sparse
confluent
b
EdU positive cells [%]
*
DMSO
Blebb
c
MCF-10A
EdU positive cells [%]
time after seeding cells [h]
sparse
confluent
d
EdU positive cells [%]
N.S.
N.S.
DMSO
Blebb
sparse
confluent

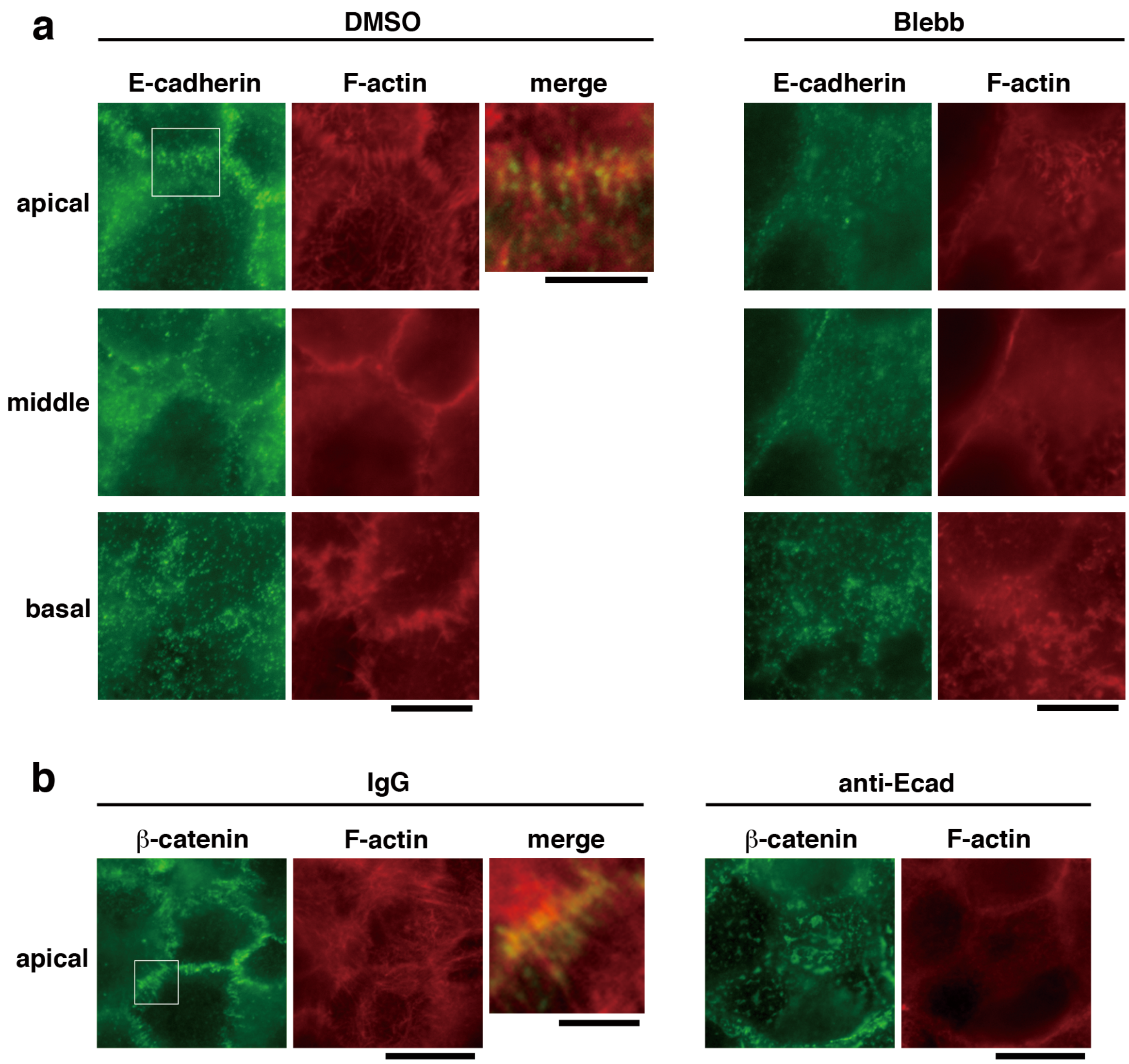
a
DMSO
Blebb
E-cadherin
F-actin
merge
E-cadherin
F-actin
apical
middle
basal
b
IgG
anti-Ecad
β-catenin
F-actin
merge
β-catenin
F-actin
apical

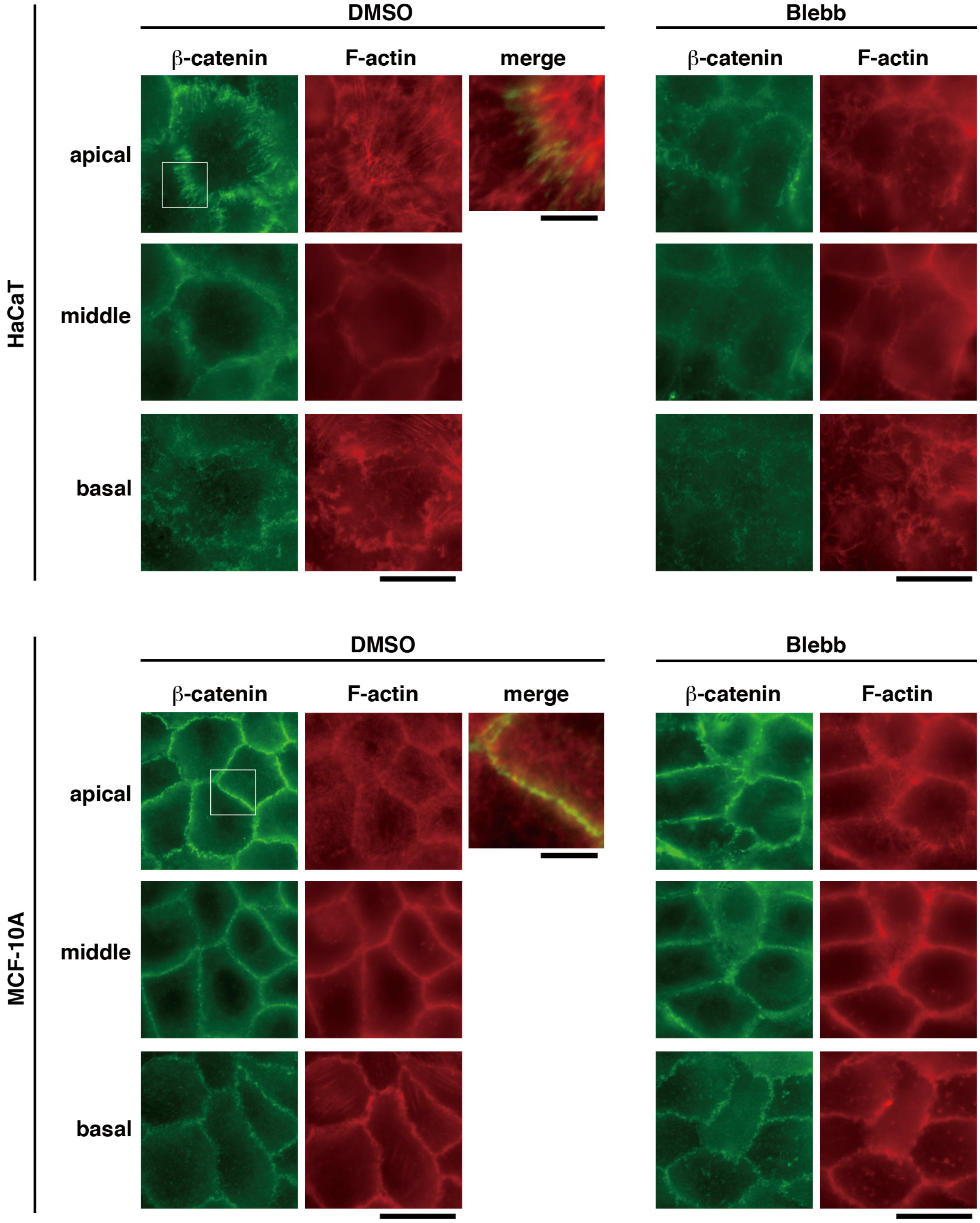

HaCaT
DMSO
Blebb
β-catenin
F-actin
merge
β-catenin
F-actin
apical
middle
basal
MCF-10A
DMSO
Blebb
β-catenin
F-actin
merge
β-catenin
F-actin
apical
middle
basal

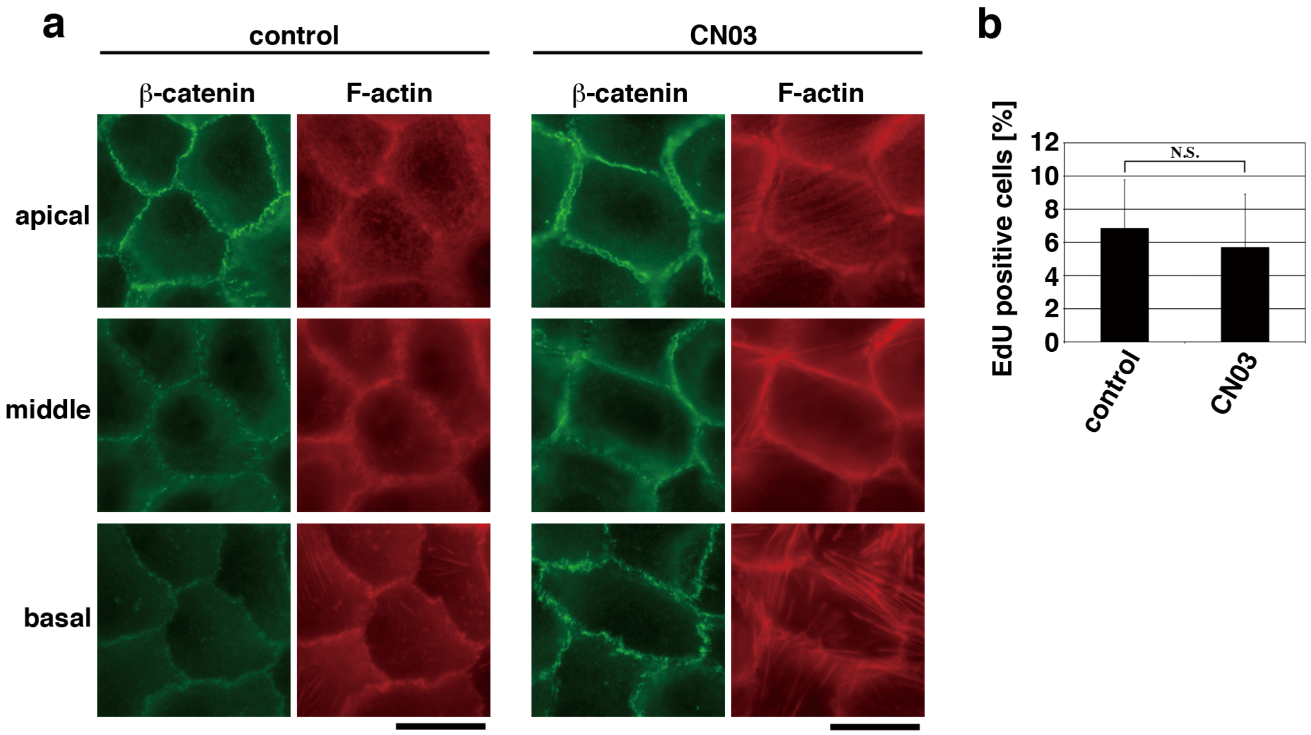

a
control
CN03
β-catenin
F-actin
β-catenin
F-actin
apical
middle
basal
b
EdU positive cells [%]
12
10
8
6
4
2
0
N.S.
control
CN03

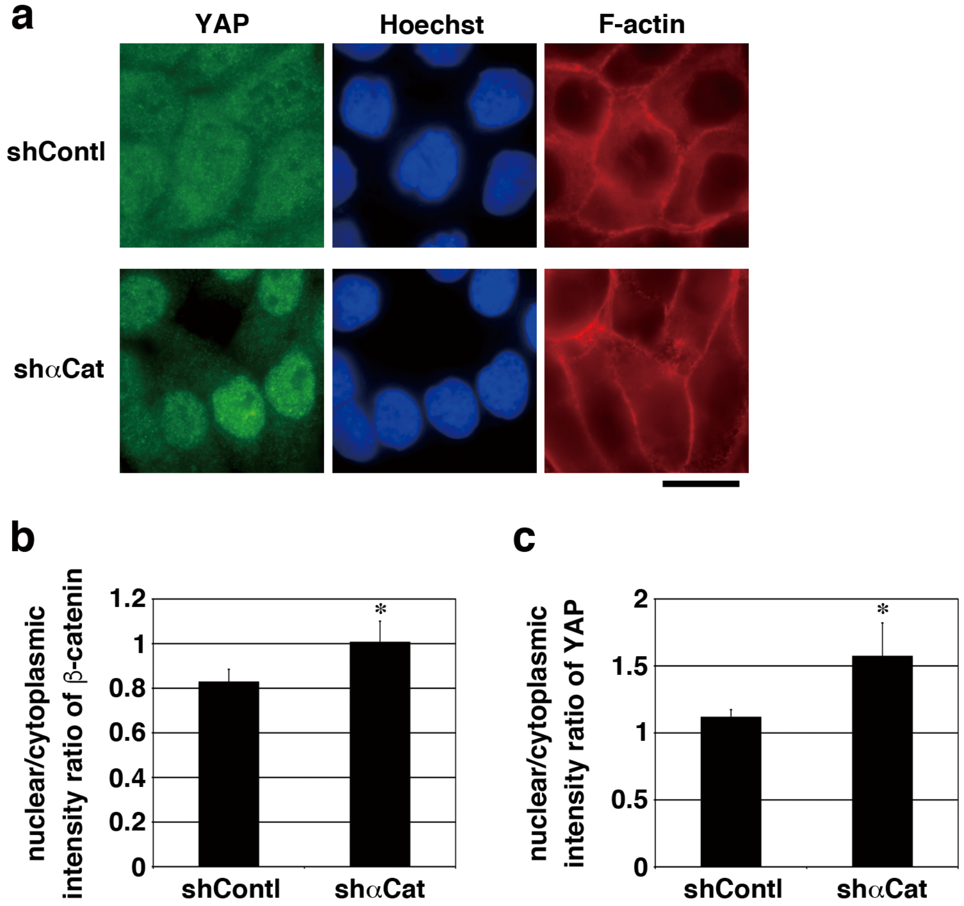
a
YAP
Hoechst
F-actin
shContl
shαCat
b
nuclear/cytoplasmic intensity ratio of β-catenin
1.2
1
0.8
0.6
0.4
0.2
0
*
shContl
shαCat
c
nuclear/cytoplasmic intensity ratio of YAP
2
1.5
1
0.5
0
*
shContl
shαCat

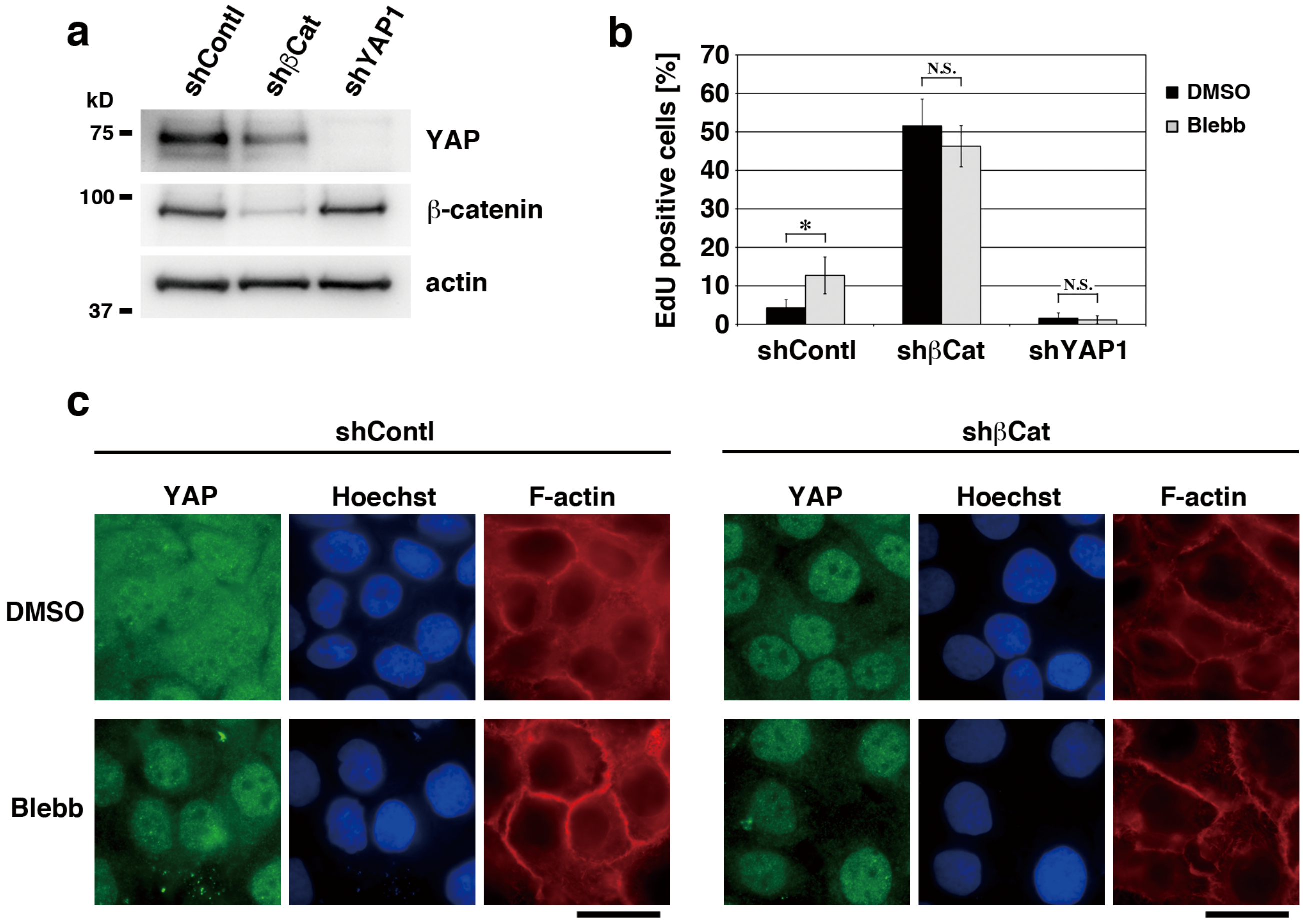
a
shContl
shβCat
shYAP1
kD
75
100
37
YAP
β-catenin
actin
b
EdU positive cells [%]
70
60
50
40
30
20
10
0
N.S.
*
N.S.
DMSO
Blebb
shContl
shβCat
shYAP1
c
shContl
shβCat
YAP
Hoechst
F-actin
DMSO
Blebb

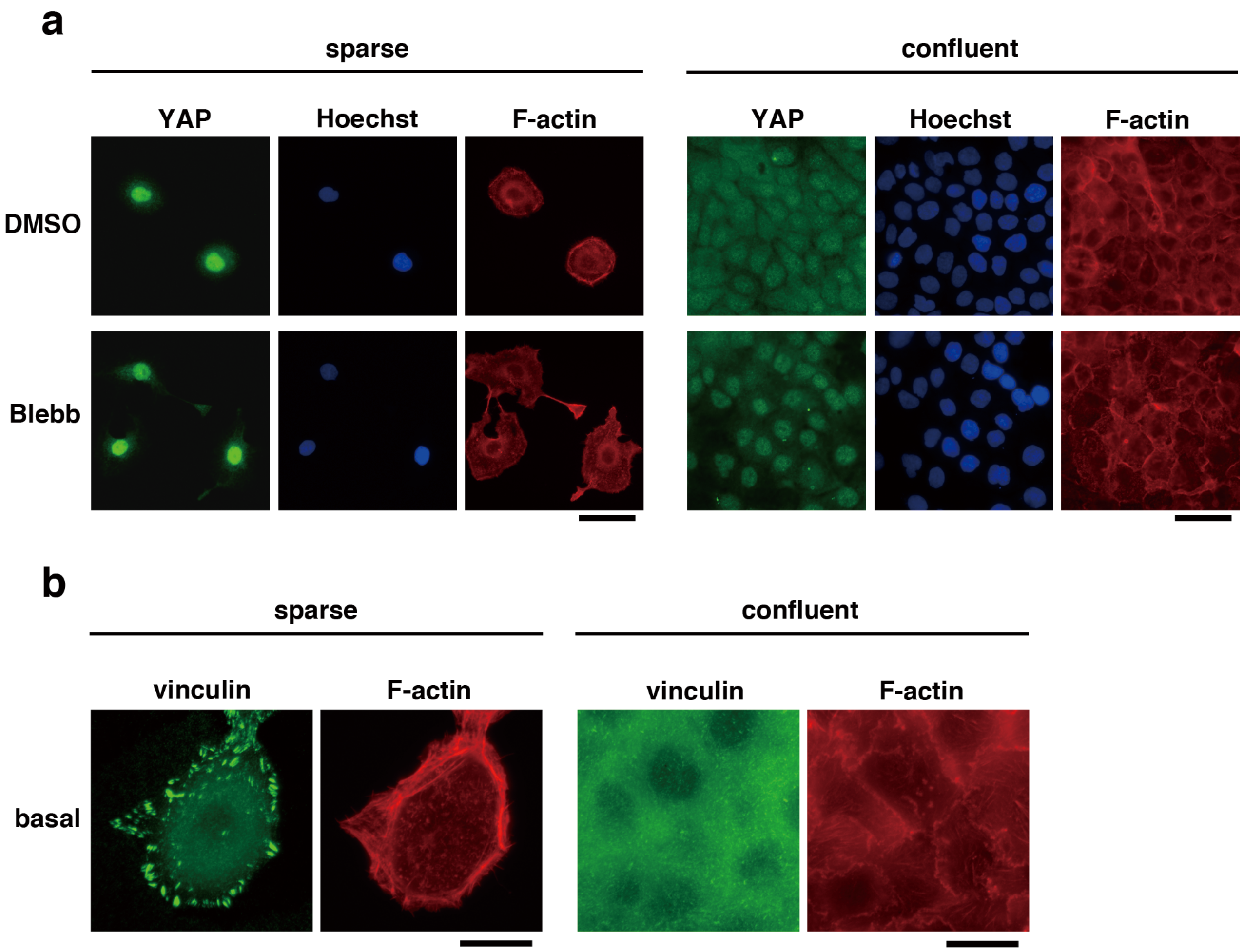
a
sparse
confluent
YAP
Hoechst
F-actin
YAP
Hoechst
F-actin
DMSO
Blebb
b
sparse
confluent
vinculin
F-actin
vinculin
F-actin
basal

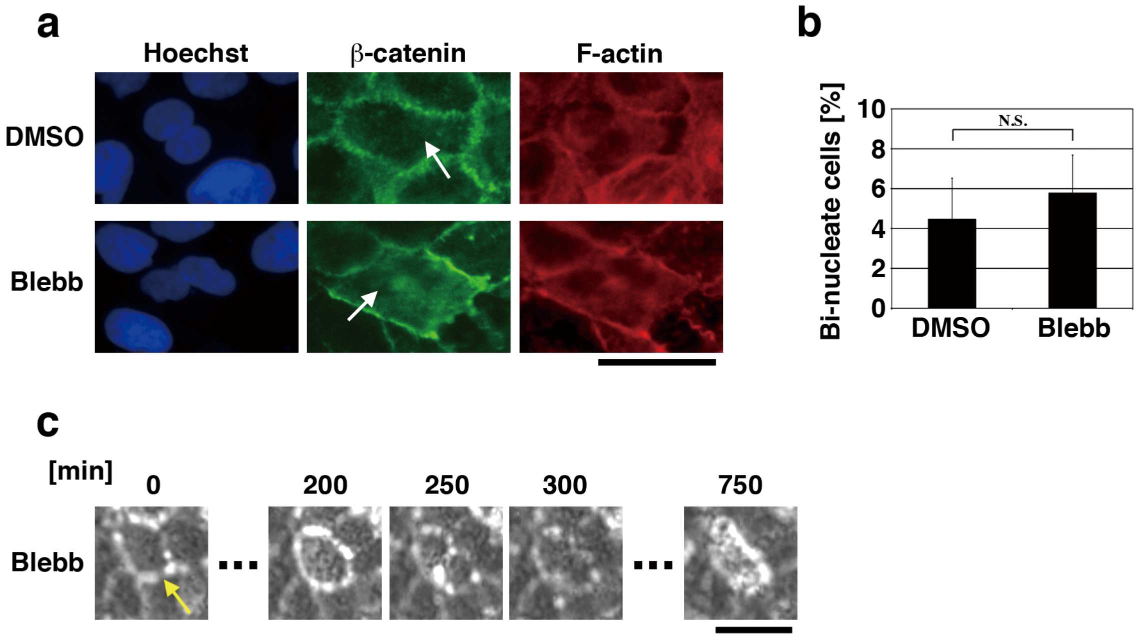

a
Hoechst
β-catenin
F-actin
DMSO
Blebb
b
Bi-nucleate cells [%]
10
8
6
4
2
0
N.S.
DMSO
Blebb
c
[min]
0
200
250
300
750
Blebb
